# Eradication rate and safety of a “simplified rescue therapy”: 14‐day vonoprazan and amoxicillin dual regimen as rescue therapy on treatment of *Helicobacter pylori* infection previously failed in eradication: A real‐world, retrospective clinical study in China

**DOI:** 10.1111/hel.12918

**Published:** 2022-07-25

**Authors:** Wen Gao, Guigen Teng, Chi Wang, Ying Xu, Yixuan Li, Hong Cheng

**Affiliations:** ^1^ GI Department Peking University First Hospital Beijing China

**Keywords:** amoxicillin, dual therapy, *Helicobacter pylori* infection, simplified rescue therapy, vonoprazan

## Abstract

**Background:**

The currently recommended quadruple regimens as rescue therapy on *Helicobacter pylori* infection were not as effective as being supposed, especially in those who had failed two or more times. Dual regimen composed of vonoprazan (a potassium‐competitive acid blocker) and amoxicillin might be an option since it's effective in eradication therapy as first‐line treatment.

**Objective:**

As a real‐world retrospective study, data were collected to evaluate the efficacy and safety of vonoprazan and amoxicillin dual regimen as rescue therapy in *Helicobacter pylori* positive patients who had failed one or more times in their previous treatment.

**Methods:**

From May 2020 to June 2021, the clinical data of patients who had failed in *Helicobacter pylori* infection treatment were collected in GI department of Peking University First Hospital, Beijing, China. Patients were given vonoprazan 20 mg or 40 mg per day and amoxicillin 3000 mg per day (VA dual therapy) for 14 days as rescue treatment. *Helicobacter pylori* status was evaluated by ^13^C‐urease breath test 6 weeks after treatment. All adverse effects during treatment were recorded.

**Results:**

A total of 186 patients were enrolled, including 67 males and 119 females. All of them had failed for 1 ~ 7 times in their previous treatment. Successful eradication was achieved in 172 patients (92.5%, 172/186). The adverse effects (referring to skin rash, abdominal pain, diarrhea, and headache), mainly mild and did not cause quit of treatment, occurred in 14 patients (7.5%, 14/186) and all symptoms relieved spontaneously.

**Conclusions:**

Dual regimen composed of vonoprazan and amoxicillin for 14 days was effective and safe as rescue therapy in *Helicobacter pylori* infection treatment. It could be chosen as a “simplified rescue therapy” with relatively high eradication rate no matter how many times the patients had failed and what regimens they had used previously.

## 1 INTRODUCTION

*Helicobacter pylori* (*H. pylori*) infection is a major risk factor for development of gastritis, peptic ulcer, and gastric cancer. Successful eradication is an effective strategy to decrease the risk of gastric cancer.^1^, ^2^ The efficacy of bismuth‐based quadruple therapy as first‐line therapy has been clearly established, which is now been estimated as the mostly widely used regimen in China. As a country in which antibiotic resistance of *H. pylori* is pretty high especially in clarithromycin, metronidazole, and fluoroquinolone, it's relatively difficult to choose treatment regimen when it comes to the patients who had failed in their previous therapies. The recent clinical guidelines did not provide accordant advices for rescue therapy according to different populations with different antimicrobial resistance status and different regimens used before. Individual treatment with antibiotics‐sensitivity test would be ideal but not easy to be widely used in clinical practice.

Different from the other antibiotics, there is consistent reports that the primary and secondary resistance rates of *H. pylori* to amoxicillin maintained at a low level.^3^, ^4^In recent years, proton‐pump inhibitor (PPI) plus amoxicillin dual therapy has gained increasing attention worldwide because of its effectiveness with a cure rate of 95.3% in first‐line treatment and 89.3% in second‐line treatment.^5^, ^6^, ^7^ It is currently believed that the outcome of the dual therapy is pH‐dependent. Routine dose of PPIs has been proven to be unable to reliably maintain the intragastric pH value at a suitable level required by amoxicillin,^8^ which might be the main reason dual therapy failed in mid‐ to late‐1990s. Vonoprazan (VPZ), a novel potassium‐competitive acid blocker, which became available in 2015, has significantly higher acid suppression effect by inhibiting the H + ‐K+ exchange directly to gain a predominant pH elevation for 24 h. Our study aimed to clarify the effectiveness and safety of VPZ plus amoxicillin dual regimen as simplified rescue treatment on the eradication of *H. pylori* infection no matter what regimens the patients had accepted in their previous treatment.

## 2 MATERIALS AND METHODS

### 2.1 Study design and participants

A real‐world, retrospective study was conducted in the Department of Gastroenterology in Peking University First Hospital, Beijing, China. Data were collected from May 2020 to June 2021. All patients who had accepted VA dual therapy as their rescue treatment were involved. The general data are shown in Table 1.The primary endpoint was the eradication rate, the secondary endpoint was the prevalence of adverse events, compliance, and related factors which might affect the cure efficacy of treatment.

**TABLE 1:** Demographic and clinical data of all patients and patients who succeeded and failed in VA dual rescue therapy

	Total (*n* = 186)	VA success (*n* = 172)	VA failure (*n* = 14)	*p* value (Chi‐square)
Age (mean, SD) year Range	48.8 (12.9) 18–73	48.6(13.0) 18–73	51.4(12.7) 28–71	_
Gender (M/F)	67/119	62/110	5/9	0.97
BMI (mean, SD) kg/m2	22.5 (3.1)	22.1(3.6)	22.9 (3.1)	0.732
A used previously	155(83.3%)	141(82.0%)	14(100%)	0.08
Smoking	17 (9.1%)	14(8.1)	3 (21.4%)	0.10
Alcohol drinking	43 (23.1%)	39(22.7%)	4(28.6%)	0.61
Family history of gastric cancer	33(17.7%)	30(17.4%)	3(21.4%)	0.71
Treatment times previously				
1	84(45.2%)	78(45.4%)	6(42.9%)	0.40
2	54(27.4%)	52(30.2%)	2(14.2%)	
≥3	48(27.4%)	42(24.4%)	6(42.9%)	
Endoscopy diagnosis				
Gastritis	152(81.7%)	141(90.0%)	11(78.6%)	0.75
Gastric ulcer	8(4.3%)	8(4.7%)	0	0.41
Duodenal ulcer	16(8.6%)	15(8.7%)	1(7.1%)	0.84
Complex (gastric and duodenal) ulcer	8(4.3%)	6(3.4%)	2(14.3%)	0.06
Gastric cancer	2(1.1%)	2(1.1%)	0	0.68
Compliance*	185(99.5%)	171(99.4%)	14(100%)	0.77

*Note*: Data are *n* (%), or mean (SD, standard deviation).

Abbreviations: A, Amoxicillin; BMI, body mass index; V, Vonoprazan; VA, vonoprazan + amoxicillin dual therapy.

*Compliance, taken >80% of tablets.

### 2.2 Diagnosis of *H. pylori* infection and treatment regimen

*H. pylori* infection was diagnosed as positive in ^13^C‐urease breath test (^13^C‐UBT)(75 mg ^13^C‐urea, Shenzhen Zhonghe Headway Bio‐Sci & Tech Co., Ltd.). As to the outcome of treatment, *H. pylori* status was determined by ^13^C‐UBT at least 6 weeks after completion of therapy.

Vonoprazan (VPZ, 20 mg/tablet, Takeda Pharmaceutical Co.) plus amoxicillin (250 mg/capsule, the United Laboratories International Holdings Limited) dual therapy (VA dual therapy) consisted of VPZ 10 mg twice daily (10 mg = half tablet with 20 mg/tablet, body weight ≤ 55 kg) or 20 mg twice daily (body weight > 55 kg) and amoxicillin 3000 mg per day (mostly 1000 mg t.i.d,750 mg q.i.d in few patients. The frequency of amoxicillin administration was given casually as t.i.d or q.i.d more than well‐designed). The treatment course was 14 days. VPZ was suggested to be taken half an hour before breakfast and dinner. Amoxicillin was suggested to be taken just after breakfast, lunch, and dinner and before sleep if q.i.d.

### 2.3 Antibiotic susceptibility test

Some of the patients had got bacteria culture and antibiotic susceptibility test. Two biopsies were collected from the gastric antrum and corpus to culture *H. pylori* strains before treatment. When a positive culture was obtained, antibiotics’ susceptibility to amoxicillin (AMX), clarithromycin (CLA), metronidazole (MTZ), levofloxacin (LEV), moxifloxacin (MOX), and tetracycline (TET) was tested using Epsilometer test (E‐test) strips (BioMerienx, France) on Columbia blood agar plates containing 8% fresh defibrinated sheep blood. After 72 h of incubation under microaerobic atmosphere, the minimum inhibitory concentration (MIC) of each antibiotic was determined.

Resistance to AMX, CLA, MTZ, LEV, and TET was defined as MIC >0.125 mg/L, MIC >0.5 mg/L, MIC >8 mg/L, MIC >1 mg/L, and MIC >1 mg/L, respectively, according to the clinical breakpoints recommended by the European Committee on Antimicrobial Susceptibility Testing for *H. pylori* (EUCAST, Breakpoint tables for interpretation of MICs and zone diameters, version 10.0, 2020, http://www.eucast.org/clinical_breakpoints/). Resistance to MOX was defined as MIC >1 mg/L according to literature reports.^9^, ^10^

### 2.4 Statistical analysis

Data collected were analyzed using IBM SPSS Statistics SPSS 20.0 software (IBM Corp.,). Continuous variables were expressed as the mean ± standard deviation, and categorical variables were expressed as numbers and percentages. The significance of the *p*‐value was defined as less than .05 in the statistical analyses.

## 3 RESULTS

### 3.1 Patients enrolled and baseline characteristics

A total of 186 patients who accepted VA dual therapy as rescue treatment were enrolled, including 67 males and 119 females. All patients had failed in their previous treatment at least one time (average 2.1 times, range 1 ~ 7 times). Among them, most had accepted treatment for one time (*n* = 84, 45.7%) or two times (*n* = 54, 27.9%). 48 patients (26.3%) failed for three or more times: 26 cases failed three times (14.0%), 13 cases failed four times (7.0%), 7 cases failed five times (3.8%), 1 case failed six times (0.5%), 2 cases failed seven times (1.0%). Most of their previous treatment regimens were bismuth‐based quadruple regimens. The antibiotics mostly used were amoxicillin, clarithromycin, metronidazole, levofloxacin, or moxifloxacin, tetracycline and furazolidone were also used in some cases.

The demographic and clinical data of the patients are shown in Table 1. Thirty three of them had family history of gastric cancer. All patients were not allergic to penicillin, as they had taken amoxicillin before without side effects or proven to be safe with a negative penicillin allergy test. Although 134 patients (134/186, 72.0%) had one or more combined diseases, most of them (120/186, 64.5%) had no combined medicine during treatment. The most often combined diseases were hypertension, hyperlipidemia, and diabetes mellitus.

### 3.2 Eradication of *H. pylori* infection

All the186 cases enrolled had completed the treatment. A total of 172 patients (172/186, 92.5%, 95% CI 87.4% to 95.7%) got successful eradication.Fourteen patients (14/186, 7.5%) failed in their VA dual therapy, all failed had used amoxicillin in their previous *H. pylori* treatment. According to demographic and clinical data of the patients, there were no significant risk factors of eradication failure including gender, BMI, smoking, or alcohol drinking status, family history of gastric cancer, endoscopy diagnosis, and previous treatment times (Table 1).

In 186 patients, 83.3% of them (*n* = 155) had used amoxicillin in their previous treatment, while 16.7% of them (*n* = 31) did not used it. All the patients who had not used amoxicillin before got a successful eradication(100%, 31/31), while only 91.0% (141/155) of the patients who had used amoxicillin before eliminated the bacteria (Figure 3). There was no statistical difference in eradication rate between the two groups (*p* = 0.08 by Chi‐square test and *p* = 0.132 by Fisher's analysis).

According to different treatment times endured before, there was little difference in eradication rate. In 186 cases involved, most of them had accepted treatment for one time (*n* = 85, 45.7%) or two times (*n* = 52, 27.9%) previously. Despite different treatment times before, the overall eradication rate was 92.5% (95% CI 87.4%–95.7%, 172/186). The eradication rate was 92.9% (95% CI 84.5%–97.1%, 78/84) in patients who had failed one time, 96.3% (95% CI 86.2%–99.4%, 52/54) in patients who had failed two times and 87.5%(95% CI 74.1%–94.9%, 42/48) in patients who had failed three or more times previously. There was no statistical difference in eradication rate in different treatment time groups (*p* = 0.403, two‐tailed significant tests).

In 186 patients, 23.1% of them (*n* = 43) had accepted vonoprazan 20 mg per day (10 mg b.i.d) with body weight ≤ 55 kg while 76.9% (*n* = 143) had accepted vonoprazan 40 mg per day (20 mg b.i.d) with body weight > 55 kg in their rescue treatment. The eradication rates of different dose of vonoprazan groups were 95.3% (95% CI 83.0%–99.2%, 41/43) in 10 mg b.i.d group and 91.6% (95% CI 85.5%–95.4%, 131/143) in 20 mg b.i.d group, respectively. There was no statistically difference between them (Figure 4 **)**.

### 3.3 MIC to antibiotics of isolated *H. pylori* strains

Twenty‐five cases had got the MIC test, most of *H. pylori* strains isolated from them were resistant to CLA, MTZ, LEV, and MOX. Resistance to AMX and TET was rare (Figure 2).

According to MIC of AMX in 25 cases, 88.0% (22 of 25) of them were susceptible to amoxicillin, while 44.0% (11 of 25) of them were super‐susceptible (MIC≤0.023 mg/L), 44.0% (11 of 25) of them were susceptible (0.023 mg/L < MIC≤0.125 mg/L), 12.0% (3 of 25) of them were resistant to amoxicillin with an MIC more than 0.125 mg/L. In three patients whose *H. pylori* strains were resistant to AMX, one failed and two succeeded in VA dual therapy.

### 3.4 Compliance and adverse events

Of all 186 patients, 185 of them (99.5%) had good compliance (taken >80% of all tablets) (Table 1). All patient who got adverse events during their treatment had completed the whole course. The patient who quitted the treatment had failed one time in his previous treatment. The reason of quitting was that he had forgotten to take the drugs on time, not for the adverse events. He did not get the MIC test. Later he got a successful eradication in his third treatment with vonoprazan + bismuth + tetracycline +furazolidone quadruple therapy.

Totally 14 patients (7.5%, 95% CI 4.3%–12.6%) endured the adverse events (Table 2). The most happened adverse events were diarrhea (three of 14) and nausea (three of 14). All adverse events were mild and did not influence the completion of therapy. In all patients who suffered adverse events during treatment, only one patient (one of 14) with diarrhea failed in her eradication. Most adverse events were mild and reversible. All adverse events were spontaneously cured without intervention except one patient who had a successful treatment suffered mild skin rash occured 2 days after the end of the treatment and recovered after anti‐allergy treatment.

**TABLE 2:** Adverse events happened

Symptoms	*N*	Gender	age	BMI	Doses of VPZ	Treatment times	Treatment continuation	Eradication
Diarrhea	3	F	39	24.6	40 mg	1	Y	N
		F	34	22.3	40 mg	2	Y	Y
		F	55	22.0	20 mg	1	Y	Y
Nausea	3	F	54	22.2	40 mg	2	Y	Y
		M	31	28.4	40 mg	2	Y	Y
		F	48	30.5	40 mg	1	Y	Y
Headache	2	F	56	26.5	40 mg	3	Y	Y
		F	59	22.1	40 mg	3	Y	Y
Tongue numbness	1	F	52	18.7	20 mg	1	Y	Y
Dry mouth	1	F	47	18.4	20 mg	1	Y	Y
Abdominal pain	1	F	47	23.8	40 mg	3	Y	Y
Abdominal pain+diarrhea	1	F	59	23.0	20 mg	3	Y	Y
Abdominal pain+abdominal distension	1	M	41	25.1	40 mg	2	Y	Y
Skin rash (2 days after end of treatment)	1	M	59	21.3	40 mg	1	Y	Y
Total	14							

Note: Adverse events happened in 14 patients (7.5%), which were mild and did not affect the continuation of therapy.

Abbreviations: BMI, body mass index (kg/m2); Doses of VPZ, total doses of vonoprazan (mg).

## 4 DISCUSSION

In our study, VA dual regimen was designed as rescue treatment used in patients who failed one or more times before, no matter what regimen they had used, including those who had used PPI + amoxicillin dual therapy. The overall eradication rate was 92.5% (95% CI 87.4%–95.7%) with minimal side effects (7.5%, 95% CI 4.3%–12.6%).

Causes of treatment failure of anti‐*H. pylori* therapy include antibiotics resistance, poor compliance of patients, low gastric pH, and high bacterial load.^11^, ^12^ The prevalence of multidrug‐resistant *H. pylori* strains is increasing, especially in cases with multiple eradication failure, which makes rescue treatment difficult.^13^, ^14^ However, since the resistance rate to amoxicillin is low even after the failure of eradication, amoxicillin can be a candidate of antimicrobial agent for the rescue therapy.^8^, ^15^, ^16^, ^17^

Beyond the traditional quadruple therapy, dual therapy, which was composed of PPI + amoxicillin, was testified to be an effective regimen used in treatment of *H. pylori* infection in recent years.^5^, ^16^, ^18^After the first report in 1989, the efficacy of AMX‐contained dual therapy was unstable and being abandoned for many years.^19^, ^20^While in recent 10 years, there were more and more studies showing that it could be pretty effective.^21^ It was believed that there were two critical variables/factors that affected the efficacy of the treatment.^21^ One was to achieve and maintain a relatively high intragastric pH value, in which the antibactericidal effect of amoxicillin would be stable to get a better bioavailability in gastric cavity. The second was the concentration of amoxicillin in stomach.^21^ Amoxicillin is a time‐dependent antibiotic, which is rapidly absorbed into plasma and then to be excreted in 6 ~ 8 h after administration. Comparing with 1000 mg twice daily, a dosage of 500 ~ 750 mg per 6 h might be more likely to maintain a higher plasma concentration.^6^, ^22^

According to the choice of acid inhibitors, since the intragastric pH value might vary according to the potency of different PPIs and ethnic difference in PPI metabolism (cytochrome P450 [CYP2C19] pharmacogenetic polymorphism)^8^, vonoprazan was chosen as part of the combination. Vonoprazan (VPZ) is the first clinically available potassium competitive acid blocker, which could provide fast and powerful acid inhibition, suggesting it might be possible to sustain a higher intragastric pH value. It was observed that a pH >4.0 status could be obtained at 4 h and to sustain for 24 h after the first administration of VPZ.^13^, ^23^ The effectiveness of VPZ and amoxicillin dual therapy used as first‐line treatment was pretty good with a eradication rates varied as 85%–90% in Japan, while there was little data on its effect on rescue treatment.^3^, ^13^ In our study, the dual therapy was composed of vonoprazan 20/40 mg per day (10/20 mg b.i.d) and amoxicillin 3000 mg per day (1000 mg t.i.d or 750 mg q.i.d). It was used as rescue therapy for patients who failed in their previous treatment, no matter how many times they had failed and what eradication regimens they had used before.

**FIGURE 1:**
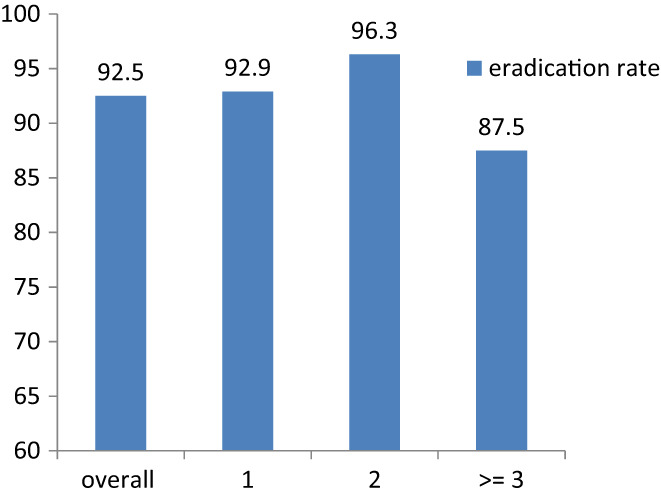
The eradication rate of VA dual treatment according to different failure times previously. All patients had endured previous treatment failure. Most of them had accepted treatment for one time (*n* = 84, 45.7%) or two times (*n* = 54, 27.9%). Forty‐eight patients (26.3%) failed for three or more times: 26 cases failed three times (14.0%), 13 cases failed four times (7.0%), 7 cases failed five times (3.8%), 1 case failed six times (0.5%), 2 cases failed seven times (1.0%). The overall eradication rate of VA dual rescue therapy was 92.5% (172/186). The eradication rate was 92.9% (78 of 84) in patients who had failed one time, 96.3% (52 of 54) in patients who had failed two times, and 87.5% (42 of 48) in patients who had failed three or more times previously

In this study, all patients had not used VA dual therapy, while some of them had used PPI + AMX dual therapy before (3/186). Altogether 186 subjects were enrolled, the eradication rate was 92.5% (172/186). Fourteen patients failed in VA dual therapy. We tried to identify factors that might be potential risk factors of eradication failure, such as gender, BMI, smoking, alcohol drinking, previous use of amoxicillin, or previous treatment failure times. It seemed that the previous use of amoxicillin and smoking might influence the eradication efficacy, but not statistically (Table 1). A recent meta‐analysis showed that although smoking increases the failure rate of *H. pylori* eradication treatment, however, when vonoprazan is used to treat the *H. pylori* infection, smoking has no effect on the eradication rate.^24^ Although it inclined the more times the patients had failed, the less possibility they succeeded in VA dual therapy, previous failure times did not influence the efficacy of rescue treatment statistically (Figure 1).

**FIGURE 2:**
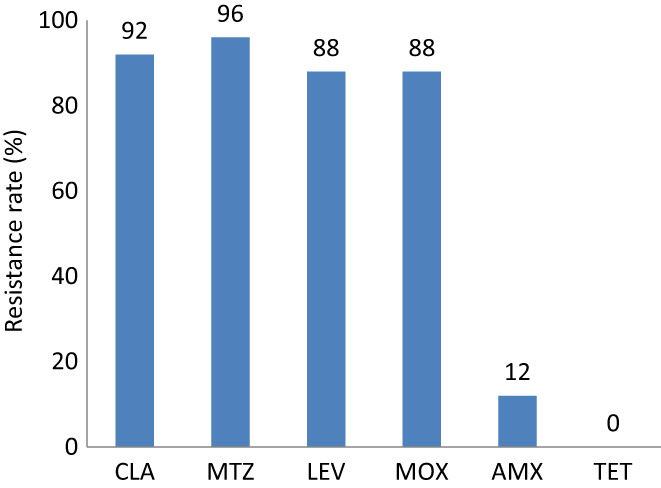
Prevalence of antimicrobial resistance of 25 cases who had been performed MIC tests. 5 cases had accepted the MIC test, most of *H. pylori* strains isolated from them were resistant to CLA (92%, 23 of 25), MTZ (96%, 24 of 25), LEV (88%, 22 of 25), and MOX (88%, 22 of 25). Three of 25 cases were resistant to AMX (12%) and none of them resistant to TET. Resistance to antibiotics was defined as: AMX resistant at MIC >0.125 mg/L; TET resistant at MIC >1 mg/L; CLA resistant at MIC >0.5 mg/L; MTZ resistant at MIC >8 mg/L; LEV resistant at MIC>1 mg/L; MOX resistant at MIC >1 mg/L. CLA: clarithromycin; MTZ: metronidazole; LEV: levofloxacin AMX: amoxicillin; TET: tetracycline; MOX: moxifloxacinMIC: Minimum Inhibitory Concentration

**FIGURE 3:**
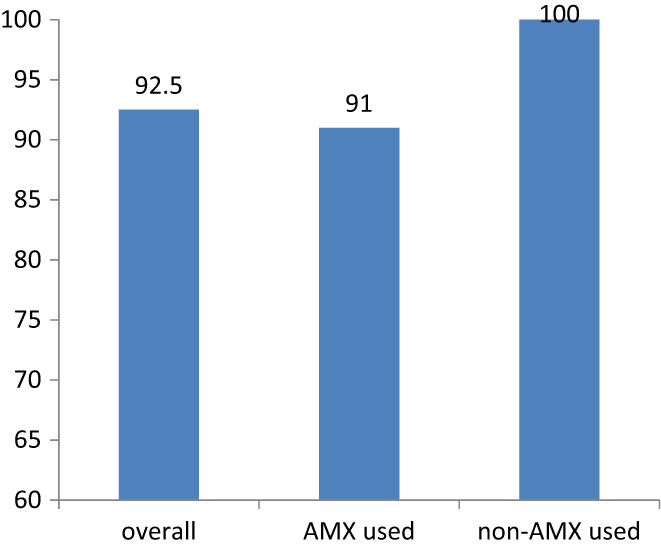
Eradication rates of patients who had or had not used amoxicillin in their previous treatment. In 186 patients, 83.3% of them (*n* = 155) had accepted previously treatments in which amoxicillin was included, while 16.7% of them (*n* = 31) did not get a regimen contained amoxicillin before. In all 186 patients, the overall eradication rate was 92.5%(172 of 186). In patients who had not used amoxicillin in their previous treatment, all of them got a successful eradication (100%, 31 of 31). While 91.0% (141 of 155) of patients who had used amoxicillin before eliminated the bacteria

**FIGURE 4:**
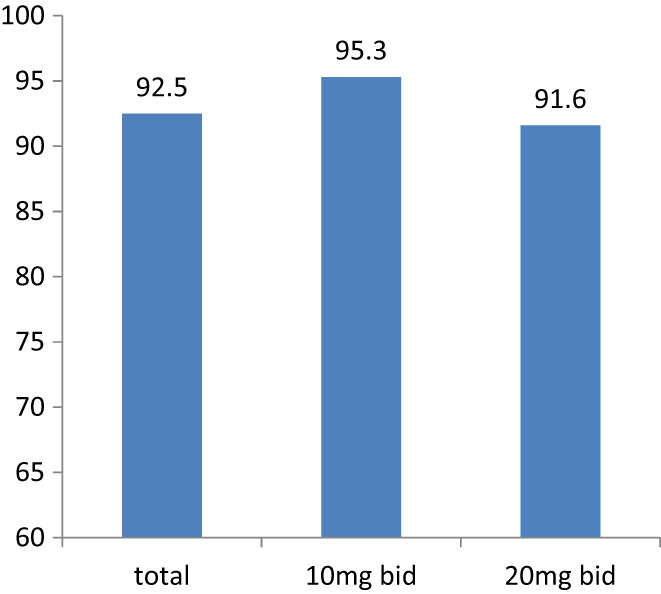
Eradication rates of total 186 patients and patients who had accepted regimens with 10 mg bid or 20 mg b.i.d vonoprazan. In 186 patients, 23.1% of them (*n* = 43) had accepted 20 mg/d vonoprazan while 76.9% (*n* = 143) with 40 mg/d vonoprazan in their rescue treatment. In all 186 patients, the overall eradication rate was 92.5% (172 of 186). The eradication rates of different dose of vonoprazan were 95.3% (41 of 43) in 10 mg b.i.d and 91.6% (131 of 143) in 20 mg b.i.d, respectively

In 25 cases who got an MIC test during treatment, three cases were resistant to AMX with an MIC of .25 mg/L (resistance defined as a MIC >0.125 mg/L). Among them, one case failed in the VA dual treatment while two of them succeeded in eradication. All 14 who failed had used amoxicillin in their previous treatment, three of them got MIC test with results of .125 mg/L, .125 mg/L and .25 mg/L to AMX, with one of three was defined as resistant to AMX theoretically. Although we did not know the detail about it, it seemed that resistance to AMX was not absolute contraindication in VA dual therapy. There was an inclination that VA dual therapy might be more effective in patients who had not used amoxicillin before.

To our knowledge, this is the first real‐world study to reveal the efficacy of vonoprazan and amoxicillin dual regimen (VA dual regimen) as rescue treatment in China. In previous studies, dual therapy with VPZ was mostly used as first‐line treatment, and the eradication rates varied as 85% ~ 90% in Japan.^13^, ^25^ In clinical practices from Japan, VPZ was mostly used as component of triple therapy in first‐line (VPZ + amoxicillin + clarithromycin), second‐line (VPZ + amoxicillin + metronidazole), and third‐line (VPZ + amoxicillin + sitafloxacin) treatment of *H. pylori* infection. VPZ‐contained triple regimens had a relatively high eradication rate as 88.1%, 80.1%, and 75.8%, respectively.^26^ Although vonoprazan appeared to restore the effectiveness of triple therapy, the improvement was almost entirely to improved effectiveness of amoxicillin dual therapy componentand resulted in the majority (>85% currently in Japan) of those receiving vonoprazan + amoxicillin plus a second antibiotic (e.g., clarithromycin, metronidazole, fluoroquinolone, or rifabutin) receiving no benefit from the second antibiotic.^27^ It seemed that the only contribution of the second antibiotic is to increase global antimicrobial resistance.^27^, ^28^, ^29^

From the data of our study, it was supposed that the VA dual therapy was effective as rescue treatment no matter what regimen had been given and which antibiotics had been used before. The VA dual therapy could be defined as a “simplified rescue therapy.”

During the treatment course of the VA dual therapy, the adverse events happened were mild and did not cause quitting or failure of treatment. The administration mode was suitable since the compliance of patients was pretty good.

There were many limitations in our study. As a real‐world retrospective study, it's not a randomized controlled trial. The regimens were not consistent, whereas components of dual therapy were the same as vonoprazan + amoxicillin, the administration frequency or doses in VA dual therapy varied. The frequency of amoxicillin was given either t.i.d or q.i.d casually more than well‐designed, although the total dose was the same as 3000 mg per day. The total doses of vonoprazan also varied (20mg or 40 mg per day) with the body weight of patient. As to the MIC analysis, there was only 25 cases who got a successful *H. pylori* culture and antibacterial susceptibility test, depending on the patients' willingness. The MIC data were limited and not very representative. Based on the results of this retrospective study, a well‐designed random controlled prospective clinical trial could be anticipated being performed in the future.

## 5 CONCLUSION

The utility of vonoprazan to replace traditional proton‐pump inhibitor (PPI) as part of components in *H. pylori* treatment, especially in cases with multiple antibiotics resistance, was effective and safe. The VA dual therapy (vonoprazan 20/40 mg per day plus amoxicillin 3000 mg per day) would be effective and safe on treatment of *H. pylori* infection no matter how many times the patients had failed or which antibiotics they had used before. The safety of the regimen and compliance of patients were pretty good. To increase the eradication efficacy, we recommend 14 days VA dual regimen as a “simplified rescue therapy” on treatment of *H. pylori* infection, especially in those who had not used amoxicillin before.

## CONFLICT OF INTEREST

The authors have no competing interests.
